# *Pediatric Neurology Briefs*: Year in Review

**DOI:** 10.15844/pedneurbriefs-35-1

**Published:** 2021-01-06

**Authors:** John J. Millichap

**Affiliations:** 1 Division of Neurology, Ann & Robert H. Lurie Children’s Hospital of Chicago, Chicago, IL; 2 Departments of Pediatrics and Neurology, Northwestern University Feinberg School of Medicine, Chicago, IL

**Keywords:** Neurology, Pediatrics, Child Development, Nervous System Diseases, Brain Diseases

## Abstract

In 2020, the mission of *Pediatric Neurology Briefs* (PNB) remains the same: “PNB is a continuing education service designed to expedite and facilitate the review of current scientific research and advances in child neurology and related subjects.”

In 2021, the mission of *Pediatric Neurology Briefs* (PNB) remains the same: “PNB is a continuing education service designed to expedite and facilitate the review of current scientific research and advances in child neurology and related subjects.” PNB has been published since 1987 and consists of 34 volumes with greater than 3700 articles and includes over 10,000 citations referencing the works of approximately 28,000 scholarly authors. PNB was relaunched as an open access, peer-reviewed journal with an expanded editorial board in January 2015. In 2018, the publishing model of PNB evolved even further. The canonical version of the article was published online as soon as it was accepted and typeset. The published articles are collected into online volumes, and an annual collection for print will be available at the end of every year.

The Editors of PNB select source articles using criteria that include recent publication in a peer-reviewed journal and a topic of clinical value to practicing pediatric neurologists. Contributing Editors provide detailed summaries of published articles, followed by commentaries based on their experience and corroborated by appropriate supplementary citations. In 2015, PNB launched a new website and content management system capable of organizing peer-review and providing improved indexing, DOI assignment, and online full-text article view. Before the internet, the Editor periodically compiled and published the PNB articles in book form with index, according to subject heading and chronological order [1-3].

In 2016, digitization of the 30 years of back issues was completed. There are now over 3700 full-text open access articles available on the journal website. Also, in 2016, PNB was selected for inclusion in PubMed Central® (PMC). PMC is a free archive of biomedical and life sciences journal literature at the U.S. National Institutes of Health's National Library of Medicine (NIH/NLM). The most highly accessed articles published in 2020 covered topics related to new epilepsy treatment [4], mechanism of the ketogenic diet [5], and genetic diagnosis [6].

Despite the immense personal and professional demands of the global COVID-19 pandemic, PNB published an increased number of manuscripts this year. The annual collection organizes the articles under 17 different sections and is available for download in PDF format here: Pediatric Neurology Briefs - Vol. 34 - 2020. The Editors would like to take this opportunity to sincerely thank the 2020 Contributing Editors for their effort and expertise (listed below in publication order):

Matheus G. Ferreira

Ethan J. Rosenberg

Abigail Schwaede

Vamshi K. Rao

Jun T. Park

Iga A. Fudyma

Joanna Garcia Pierce

Rifali Patel

Sameh S. Morkous

Peter Penzes

Lalitha Sivaswamy

Nancy L. Kuntz

Mark S. Wainwright

Darius Loghmanee

Tracy S. Gertler

Lalitha Sivaswamy

Gurpreet Khaira

Daniel A. Freedman

Hesham T. Ghonim

Jena Krueger

Safiullah Shareef

Sue J Hong

Hélio A. G. Teive

Jonathan E. Kurz

Amber N. Buehner

Andrea C. Pardo

Guadalupe Fernandez-Baca Vaca

Nitin R. Wadhwani

Divakar S. Mithal

Jamie K. Capal

Marc P. Forrest

Mitchell T. Williams

Abigail N. Schwaede

Michael Cronin

Innessa Donskoy

Bridget R. McGowan

Tuhina Govil-Dalela

Jason Coryell

Jonathan E. Kurz

Jorge Vidaurre

Arayamparambil C. Anilkumar

Abhinaya Ganesh

Elizabeth Mayne

In 2021, the Editor, Dr. John J. Millichap, is thankful for the assistance from the Associate Editors, Dr. Andrea Pardo and Dr. Stephen Nelson. Topic experts interested in contributing to PNB as authors or reviewers may contact the Editor at *j-millichap@northwestern.edu.*

## Disclosures

The author has declared that no competing interests exist.

## References

[1] Millichap JG (1991). Progress in pediatric neurology.

[2] Millichap JG (1994). Progress in pediatric neurology II.

[3] Millichap JG (1997). Progress in pediatric neurology III.

[4] Pierce JG, Mithal DS (2020). Fenfluramine: New Treatment for Seizures in Dravet
Syndrome. Pediatr Neurol Briefs.

[5] Pardo AC (2020). Ketogenic Diet: A Role in Immunity?. Pediatr Neurol Briefs.

[6] Coryell J (2020). Neurologic Features with Pathogenic Copy Number
Variants. Pediatr Neurol Briefs.

